# Delayed Presentation of Shoulder Tuberculosis

**DOI:** 10.1155/2018/8591075

**Published:** 2018-09-24

**Authors:** Majid Darraj

**Affiliations:** Department of Internal Medicine, Jazan University, Jazan, Saudi Arabia

## Abstract

Infections caused by *Mycobacterium tuberculosis* (MTb) have a global distribution, with infections occurring most frequently in persons residing in or who have resided in developing nations. Pulmonary tuberculosis (Tb) is the most common form of infection caused by MTb. Osteoarticular Tb is a far less common condition than pulmonary Tb and is frequently overlooked in the differential diagnosis of persons with joint pathology. Osteoarticular Tb infections are far less common than pulmonary Tb and are usually not considered in the differential diagnosis. We describe a case of a 57-year-old immigrant African male who presented with 5 years of right shoulder pain and a restricted range of movement. Magnetic resonance imaging (MRI) concluded right shoulder septic arthritis, for which he underwent operative drainage and debridement was undertaken. The thick purulent joint fluid subsequently yielded MTb, establishing the diagnosis of osteoarticular Tb. We conclude that Tb should be suspected in cases of long-standing joint pain and stiffness, particularly in persons from endemic areas with Tb as well as patients with a previous history of Tb exposure.

## 1. The Case

**Figure 1:**
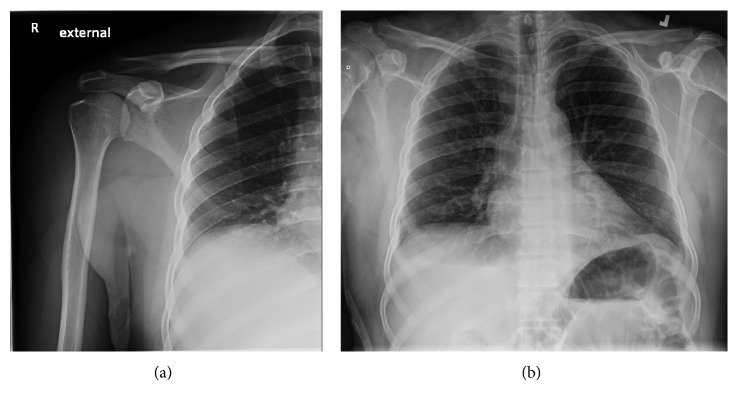
Right shoulder and chest XR showing prominence of the cephalad portion of the lateral end of the clavicle suggesting the presence of an old clavicular injury in this location. The acromioclavicular and glenohumeral joints are intact. No acute bone or joint space abnormality is demonstrated. A left-sided PICC catheter is present terminating near the atriocaval junction. No complication of catheter insertion is present. The heart size is normal. The mediastinum and hila are normal. No pleural abnormality is present. The lungs are clear.

**Figure 2:**
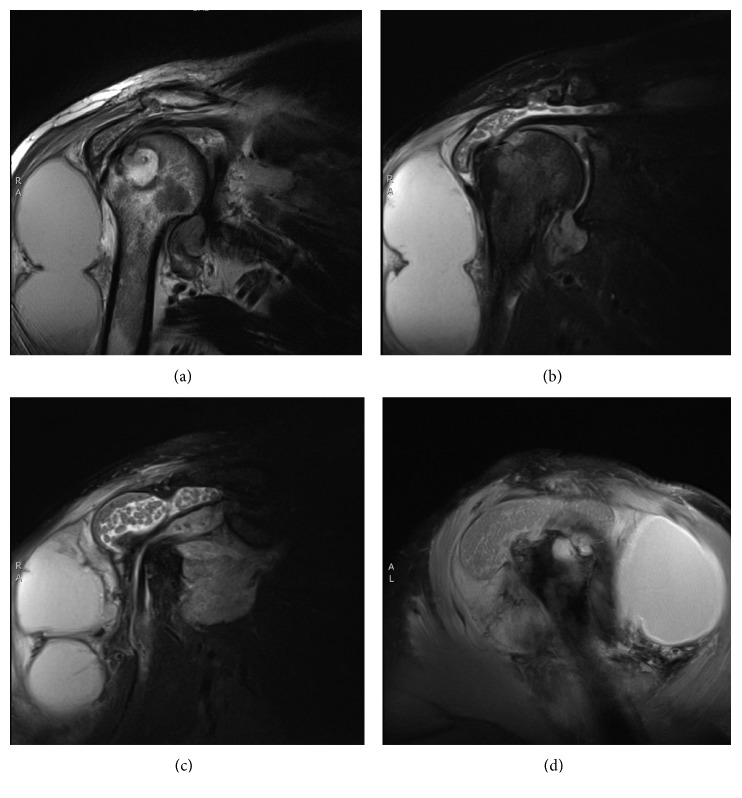
MRI of the right shoulder showing large subacromial subdeltoid fluid collection with the so-called rice bodies. A large extra-articular fluid collection is seen possibly communicating with the subacromial subdeltoid bursa.

The 57-year-old was an African male, originally from Rwanda, from where he initially immigrated to Russia. He lived in Russia for 20 years (1984–2004) before he eventually immigrated to Canada in 2004. He started to experience right shoulder pain and stiffness in 2010. There was no history of trauma to the joint and morning stiffness nor involvement of other joints. The right shoulder pain and stiffness progressed in severity over the ensuing three years and became debilitating, limiting his ability to work in construction. He had undergone multiple courses of physiotherapy and had been prescribed high doses of nonsteroidal anti-inflammatory medications without a meaningful response. For the six months prior to presentation, he had been experiencing night sweats without accompanying fever, weight loss, or respiratory symptoms. He did give a history of exposure to MTb while living in Russia as one of his workmates developed pulmonary Tb; however, our patient was not investigated at that time. Examination of the right shoulder revealed minimal swelling and that erythema and warmth were absent, but there was a decreased range of motion. Due to the worsening right shoulder pain and stiffness, a plain radiograph of the chest and shoulder was performed, revealing only an old clavicular injury and that the acromioclavicular and glenohumeral joints were intact. Neither acute nor chronic parenchymal lung, bone, or joint space abnormalities were observed. An X-ray (Figure 1) and MRI (Figure 2) of the right shoulder were therefore performed, demonstrating intra-articular effusion and erosions of articular margins, compatible with a septic joint. The erythrocyte sedimentation rate (ESR) was 68, and HIV test result was negative. Operative drainage and debridement were undertaken, and the thick purulent joint fluid subsequently yielded MTb by direct smear examination with Ziehl-Neelsen staining and nucleic acid amplification tests (NAATs), the Xpert MTB/RIF test, establishing the diagnosis of osteoarticular Tb. Antituberculosis therapy was therefore initiated with rifampin, isoniazid, pyrazinamide, and ethambutol (RIPE) in addition to pyridoxine. The subsequent Tb culture revealed no resistance to the standard first-line regimen RIPE. He was treated with RIPE for 2 months followed by rifampin and isoniazid for 10 months.

## 2. Discussion

Osteoarticular Tb is far less common than the pulmonary form. It constitutes around 1–3% of patients with extrapulmonary Tb, with 50% of cases occurring in the spine. The incidence of tuberculosis of shoulder joint is rare and accounts for only 1–2.8% of cases of skeletal Tb [1, 2]. There is usually pulmonary involvement with skeletal Tb, as was the case with our patient [3]. The infection to the joint could occur via hematogenous spread or from an adjacent infected bone. The spread from adjacent bone infection is considered to be the most common route of transmission for osteoarticular Tb [4, 5].

Positive Ziehl-Neelsen staining and/or a positive culture of MTb from the affected bone or joint is the golden standard for diagnosing osteoarticular Tb. The Xpert MTB/RIF test is highly accurate for Tb detection and offers early identification of rifampin resistance [6]. ESR could be high, as in our patient. However, across reported cases and case series of biopsy-proven osteoarticular Tb, there is no consistency of ESR [1, 2, 4, 7]. Tb in the shoulder could be of three types: (a) dry type, or *caries sicca*; (b) fulminating type, or *caries exudata*, associated with cold abscess or sinus formation; or (c) mobile type, where a passive range of motion is preserved, while variable restriction on active movement is noted due to distraction of the joint (Table 1) [3, 7]. Our patient had the *caries exudata* type.

The destructive pattern on the radiological images was used to build up the differential diagnosis for this case, which included: pyogenic osteoarthritis; salt-related arthritis, and proliferative disorders such as synovioma and sarcoma. Radiological diagnostic images (X-ray, CT scans, and MRI scans) have poor specificity for diagnosing osteoarticular Tb. However, they can be used to monitor the already-diagnosed osteoarticular lesions with a good level of precision [3]. Due to the rarity of Tb in the shoulder, accidental oversight of this rare diagnosis is expected.

Treatment strategies for this condition are primarily antimicrobial; however, surgical therapy could be used (Table 1). The optimal duration of therapy for treatment of osteoarticular Tb remains controversial (Table 2) [3, 4]. This uncertainty about the duration could be attributed to the rarity of the disease, or it could be that the spectrum of osteoarticular damage can vary; hence, the relapse rate could vary as well (Table 3). All surgical options, including partial synovectomy, decompression, abscess drainage, and debridement of infected material, must be restricted to joints with severe cartilage destruction, large abscesses, joint deformity, multiple drug resistance, or atypical mycobacteria. Our patient received a medical treatment, with the intention for this to continue for 12 months. He also underwent debridement and drainage for the effusion.

## 3. Conclusion

Nonspinal osteoarticular Tb is a rare disease and yet to be suspected in cases of unexplained long-standing joint pain and stiffness particularly with persons from endemic areas with Tb as well as in patients with a history of previous Tb exposure. The images usually used to assess the joint diseases are not specific for Tb arthritis, and hence, further histological and microbial tests are required to make the diagnosis. Osteoarticular tuberculosis should be suspected in cases of long-standing pain and restriction of movements of shoulder joint like in our patient.

## Figures and Tables

**Table 1:** Three types of Tb shoulder.

Type I: “caries sicca”	Marked wasting of the shoulder. Painful restriction of all movements.
Type II: “caries exudata”	Swelling of the joint, cold abscess. Sometimes a sinus.
Type III: “caries mobile”	Restriction of active movements of the shoulder. Nearly full passive abduction.

**Table 2:** Duration of treatment for osteoarticular Tb.

Organization	Total duration	Medical treatment recommended
World Health Organization	6 months	“RIPE” for two months followed by four months of therapy with isoniazid and rifampicin
American Thoracic Society	9 months	“RIPE” for the first two months followed by seven months of therapy with isoniazid and rifampicin
Canadian Thoracic Society	6 to 12 months	“RIPE”

RIPE: rifampicin, isoniazid, pyrazinamide, and ethambutol.

**Table 3:** Relapsing rate varies with the duration of treatment for osteoarticular Tb.

Treatment duration	Relapse rate (%)
6 months	1.35
6–12 months	0.86
>12 months	0.5

Adopted from Canadian Thoracic Society.
